# Health systems strengthening through surgical and perioperative care pathways: a changing paradigm

**DOI:** 10.1136/bmjgh-2024-015058

**Published:** 2024-11-07

**Authors:** Sivesh Kamarajah, Adesoji O Ademuyiwa, Rifat Atun, Alarcos Cieza, Fareeda Agyei, Dhruva Ghosh, Jaymie Claire Ang Henry, Souliath Lawani, John Meara, Ben Morton, Kee B Park, Dion G Morton, Teri Reynolds, Abdul Ghaffar

**Affiliations:** 1 NIHR Global Health Research Unit on Global Surgery, University of Birmingham, Birmingham, UK; 2 Surgery, University of Lagos, Akoka, Lagos, Nigeria; 3 Harvard University, Cambridge, Massachusetts, USA; 4 Department of Noncommunicable Diseases, World Health Organization, Geneve, Switzerland; 5 Department of Surgery, Komfo Anokye Teaching Hospital, Accra, Ghana; 6 Department of Paediatric Surgery, Christian Medical College, Ludhiana, India; 7 Surgery, Baylor College of Medicine, Houston, Texas, USA; 8 University of Abomey-Calavi, Cotonou, Benin; 9 Plastic and Oral Surgery, Boston Children's Hospital, Boston, Massachusetts, USA; 10 Liverpool School of Tropical Medicine, Liverpool, UK; 11 Malawi-Liverpool-Wellcome Trust Clinical Research Programme, Blantyre, Malawi; 12 Program in Global Surgery and Social Change, Department of Global Health and Social Medicine, Harvard Medical School, Boston, Massachusetts, USA; 13 Integrated Health Services, World Health Organization, Geneva, Switzerland; 14 Department of Community Health Sciences, The Aga Khan University, Karachi, Pakistan

**Keywords:** Health systems, Surgery

## Abstract

Global health has traditionally focused on the primary health development with disease-specific focus such as HIV, malaria and non-communicable diseases (NCDs). As such, surgery has traditionally been neglected in global health as investment in them is often expensive, relative to these other priorities. Therefore, efforts to improve surgical care have remained on the periphery of initiatives in health system strengthening. However, today, many would argue that global health should focus on universal health coverage with primary health and surgery and perioperative care integrated as a part of this. In this article, we discuss the past developments and future-looking solutions on how surgery can contribute to the delivery of effective and equitable healthcare across the world. These include bidirectional integration of surgical and chronic disease pathways and better understanding financing initiatives. Specifically, we focus on access to safe elective and emergency surgery for NCDs and an integrated approach towards the rising multimorbidity from chronic disease in the population. Underpinning these, data-driven solutions from high-quality research from clinical trials and cohort studies through established surgical research networks are needed. Although challenges will remain around financing, we propose that development of surgical services will strengthen and improve performance of whole health systems and contribute to improvement in population health across the world.

**Summary box**

- Perioperative care pathways are an important component in strengthening health systems, including for the Global South.
- Development of perioperative pathways, including surgery, is especially needed to improve equity of access to safe, effective care for non-communicable disease.
- Several opportunities are available to integrate surgery better into health systems through integrating surgical and chronic disease pathways.
- The global surgical and perioperative care research community can provide evidence to evaluate initiatives and implementation in health system strengthening in terms of patient outcomes.

## Background

**Figure 1:**
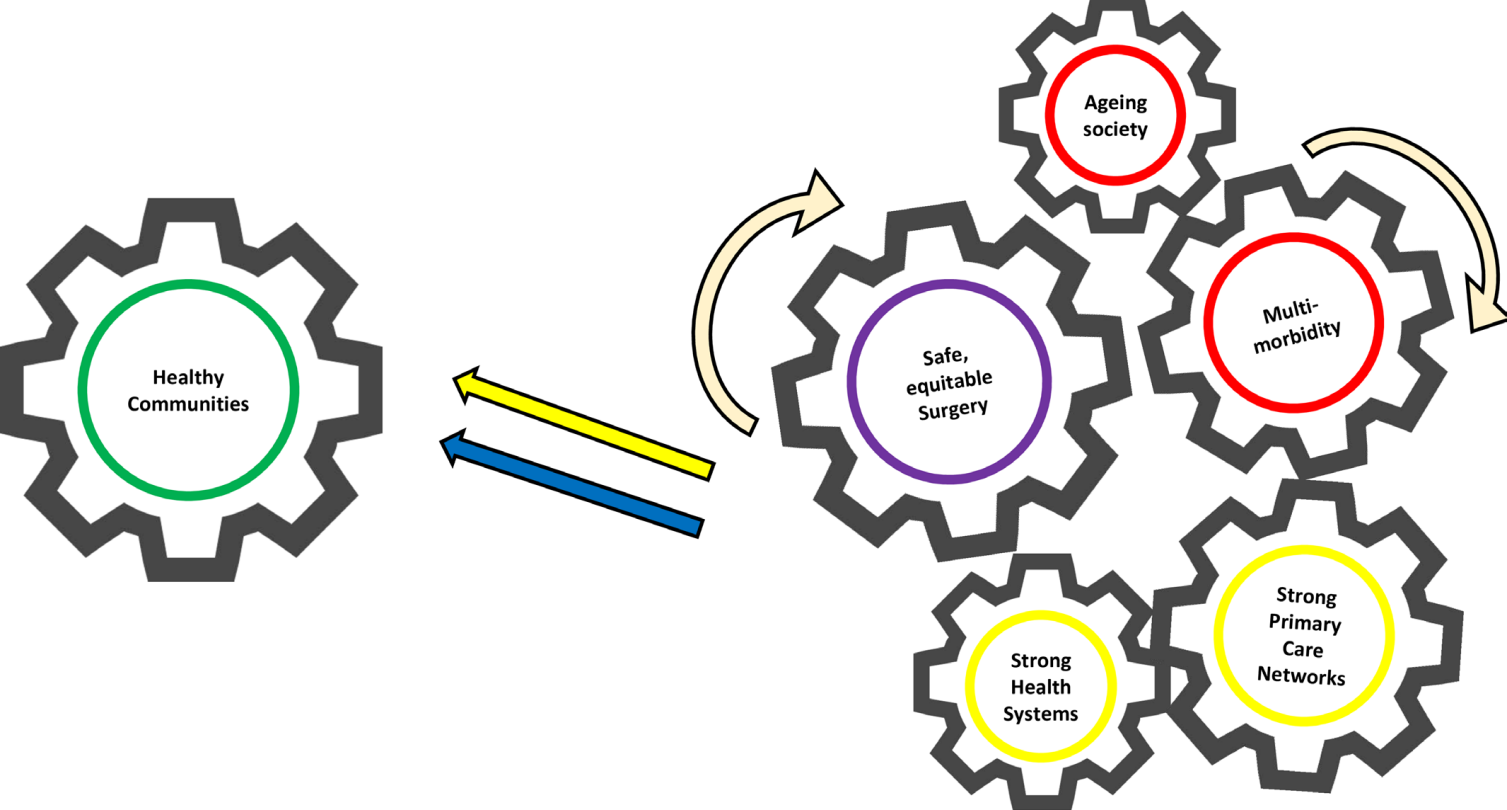
Integration of surgical pathway within wider healthcare systems.

 Access to timely, safe and affordable surgical and perioperative care has started to move to the centre stage of universal health coverage (UHC) in the recent years.^1 2^ Historically, global health focused on vertical cost-effective interventions that were convenient to the government and funders, rather than comprehensive care matched to the disease burden of communities’.^3^ However, today, many would now argue that strengthening surgical pathways could benefits systems as a whole for the community and hospitals. This will address the outstanding challenges of the 2030 sustainable development goals (SDGs), most notably 1, 3, 8, 9, 10, 16 and 17.^4^ In this article, we contend that limited development of surgical healthcare systems has seriously held back the delivery of effective and equitable healthcare across the world.^5^ We propose that investment in surgical pathways will improve performance of wider health systems and population health globally (figure 1). Notably, the importance of incorporating surgery into emergency and critical care systems is now being recognised in recent recommendations and resolutions of the World Health Assembly (WHA).^6^ Incorporation of surgery into wider health system development, notably NCDs, should now be a priority.

Delays in surgery may be due to access or affordability for the patient (or the population). All these delays are costly, resulting in advanced disease or emergency presentations, incurring substantially higher morbidity, healthcare costs and a fivefold increase in surgical mortality.^7^ Early surgery, by contrast, is safer surgery, but requires pathway integration into the continuum of care and not least into primary care. An efficient prehospital system could reduce delays in medical as well as surgical treatment. Community-based rehabilitation services have started to show patient benefit^8^ and could be further strengthened by community services supporting postsurgical recovery. An operation is at the core of surgical pathways, but it also represents a clearly defined timepoint. This point of patient contact can be used to promote community health services, address multimorbidity, as well as return people back to full socioeconomic productivity. For example, care for persons with diabetes will only be effective if surgery to treat diabetic leg ulcers is integrated. Such surgical services strengthen pathways from the community and back to promote early diagnosis/prevention, as well as improved rehabilitation after hospital care. Surgery is thereby a mechanism for whole pathway strengthening at the level of health systems.

The recent COVID-19 pandemic has both highlighted and accentuated the global need for preparedness and resilience for safe emergency, critical and operative care.^9^ The fragility of the surgical care pathway made these patients especially vulnerable both in the emergency and elective setting. Huge delays occurred across healthcare, with backlogs of elective surgery persisting into 2024.^10 11^ This created a wave of acute presentations overburdening the emergency services. Breakdown of emergency surgery has an adverse effect on all components of acute medical services from maternity health to trauma to sepsis. Elective surgical services are required to sustain working-age patients suffering from a wide range of NCDs, from cancer to inguinal hernia, preventing advanced emergency presentation and enabling a return to a full and active life. The Lancet Commission on Global Surgery^12^ estimated the need for an additional 143 million surgical procedures to save lives and prevent disability. Nearly half of these procedures are owed to children below the age of 15, emphasising the role of surgery in sustainable health for future populations. This article and those accompanying it provide an overview of where we have reached and what future action should be considered.

### What has been achieved

In 1980, the previous Director General of the WHO, Halfdan Mahler emphasised most of the world’s population lacked access to skilled surgical care, highlighting a grave social inequity in healthcare. Despite his plea, little changed in global public policy. This was because investment in surgical care was expensive and neither identified as a priority or cost-effective to health systems. Importantly, surgery not being easily assigned to vertical disease-based interventions.^3^ In 2008, there were growing calls from the global surgical community highlighting surgical care as a neglected component of global health.^13^ These early calls marked the beginning of a renewed focus on addressing the disparities in access to surgical services and pathways on a global scale. In 2015, the World Bank’s publication of the Disease Control Priorities, Third Edition (DCP3) with a dedicated volume on surgery; and The Lancet Commission on Global Surgery^12^ emphasised the urgency of addressing problems in surgical pathways globally. This major commission identified that a staggering 5 billion people lacked access to safe, affordable and timely surgical and anaesthesia care.^14^ The WHA resolution 68.15 marked the first time the WHO acknowledged strengthening surgical care systems is important to improve equitable healthcare delivery.

**Figure 2:**
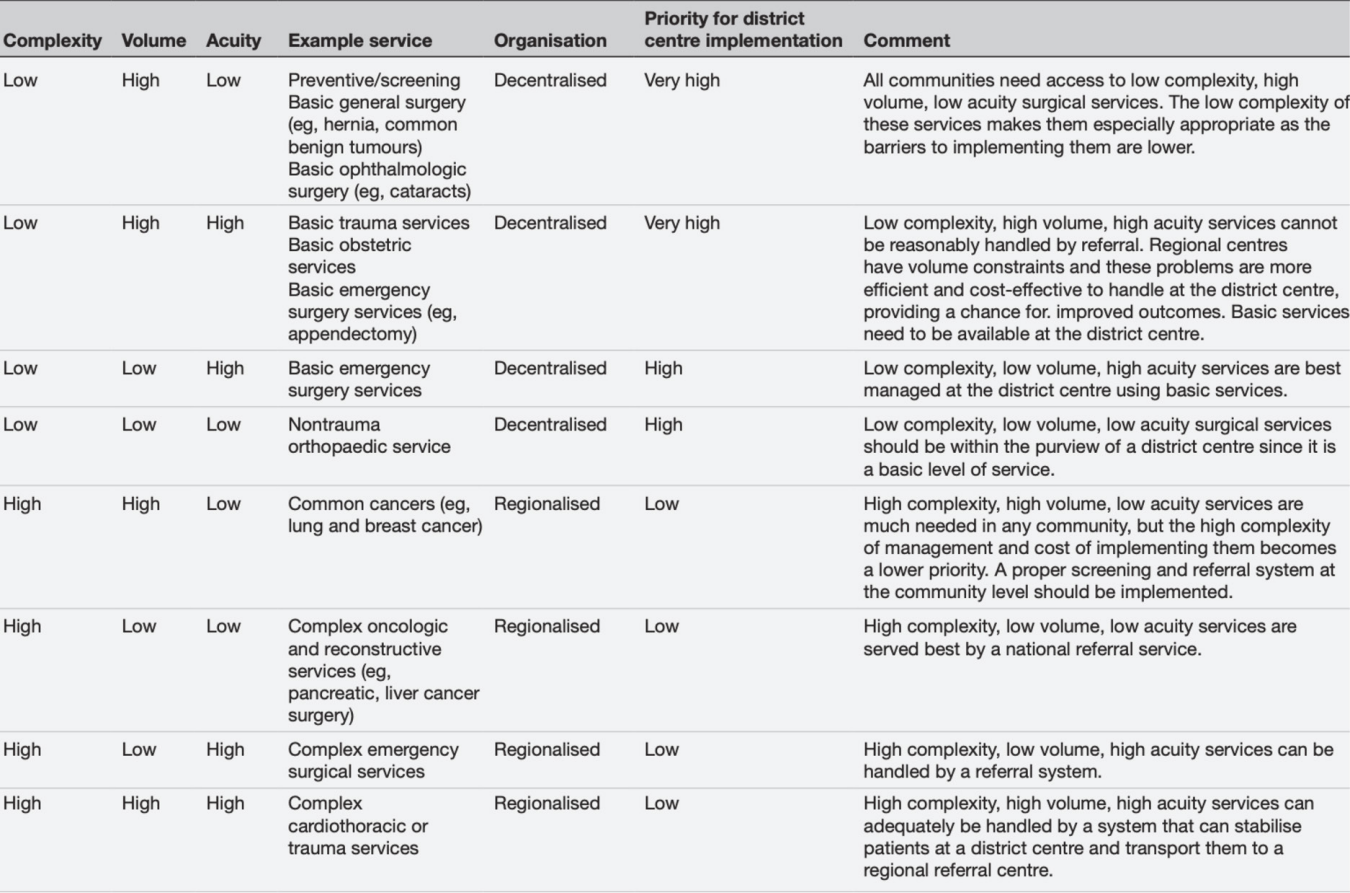
Key evidence for trauma and surgical services in low- and middle-income countries.

In the subsequent 8 years, the global surgery community witnessed a transformative period. The World Bank began publishing the key surgical indicators, providing the first-ever surgical data in the World Development Indicators. Global surgery gained a consistent presence at the WHA meetings in Geneva, and the World Health Summit incorporated global surgery into its annual agenda. Collaboratives within surgery and perioperative care, such as GlobalSurg,^7 15^ CovidSurg^16 17^ and the NIHR Global Health Research Unit in Global Surgery,^18^ facilitated global collaboration among thousands of clinicians for equitable research efforts. Countries and regions responded by developing their National Surgical Obstetric and Anesthesia Plans (NSOAPs),^19 20^ with the South African Development Community region leading a remarkable effort across 16 countries that prompted other regions, including the Pacific Islands and Latin America, to follow suit. Ecuador, under the leadership of VP Borrero, recently launched its NSOAP, becoming the first country in Latin America to embrace this crucial step towards equitable surgical care.^21^ Global Alliance for Surgical, Obstetric, Trauma, and Anaesthesia Care (G4 Alliance), a network of more than 80 global surgical organisations representing over 160 countries, has observed that context-appropriate evidence base for surgery to inform national policies and strengthen health systems remains underdeveloped. The group summarised the best level evidence on interventions geared towards increasing the quality and safety of global surgical service delivery. Data from systematic reviews,^22^,^24^ a framework for appraising these articles^25^ and a Delphi across 27 low-income and middle-income countries (LMICs) surgical providers,^26^ formed the basis of 11 Best Practice Recommendations^25^ with recommendations on the optimal organisation of surgical services (figure 2). Further developing a high-quality up-to-date evidence base for surgical service development in LMICs remains a priority today. The emerging surgical research networks are well placed to measure the patient-level benefits (or lack thereof) from this health service strengthening.

### What might be the solutions?

#### Integrating care through improved pathways

Multiple long-term health conditions or multimorbidity, defined as the presence of two or more chronic conditions,^27^ is an emerging challenge for international health systems and provides an example of the increasing need for integrated care systems.^28 29^ This is of particular importance with the rising prevalence of ischaemic heart disease, diabetes and hypertension, where primary care in LMICs is often positioned towards child and maternal health and poorly resourced to respond to the needs of rapidly ageing populations.^30 31^ Therefore, there is a high reliance on secondary hospital care for the prevention of death and disability from both communicable and non-communicable diseases (NCDs).^32^ Herein, we discuss the bidirectional opportunity for improving these pathways.

First, the index surgical presentation, both in elective and emergency settings, represents a unique opportunity to improve diagnosis and management of chronic diseases. This allows the surgical and perioperative pathway to address the patient holistically, beyond the surgical disease, thus improving access to UHC and improvements in care.^3^ This process allows improved short-term perioperative outcomes, but also improved long-term outcomes for patients through an integrated approach into primary care. It is a cross-cutting intervention that has the capability to strengthen across the breadth of health systems. It can thereby support whole systems strengthening and be used as an objective measure of the impact from such innovation. Examples of this approach can be drawn from other areas such as concurrent management of diabetes and hypertension in patients with HIV.

A recent prospective cohort study identified that the three most common chronic conditions in African surgical patients were hypertension (16.3%, 1863/11 422), HIV infection (11.0%, 1253/11 422) and diabetes mellitus (6.8%, 776/11 422).^33^ However, the clustering and interaction of chronic disease are poorly described in this context, a finding mirrored in the medical literature.^34^ The differentiation between primary conditions such as hypertension, HIV and diabetes and secondary complication conditions such as stroke, chronic kidney disease and heart disease is important to draw as interventions designed to improve the control of primary diseases could significantly reduce the medium-term and long-term risk of secondary complications, disability and death for surgical patients. In the elective surgical pathways, there is growing evidence that the elective waiting list should be considered as the ‘preparation list’,^35^ to acknowledge this expanding role. Although implementation of standard operating procedures to improve chronic disease management for elective surgical care patients is well described in high-income settings, this is less well established in sub-Saharan Africa.^36^ For patients who require emergency surgery, risk mitigation strategies are required to recognise patients with chronic disease to reduce the risk of perioperative complications. Here, a key gap is the provision of critical care services to monitor and manage patients in the immediate postoperative period. Holistic approaches to combine emergency, surgical and critical care capacity building^37^ in low-resource settings are key to drive improved outcomes for these vulnerable patients.

In this way, provision of surgical services will strengthen wider health systems (strengthening pathways from primary to secondary care as well as strengthening the continuum of care from health promotion and prevention to treatment and rehabilitation) as well as enabling better management of multimorbidity in the surgical patient. These wider health benefits must be considered when the cost of surgical services come under consideration.

#### Improving access to elective surgery

There is a need to strengthen surgery within current NCD pathways. This means patients with ischaemic heart disease or osteoarthritis requiring coronary artery bypass graft or hip replacements should be able to have timely access and safe surgery to them, respectively. In improving access to surgical care for these conditions, several areas need to be addressed in addition to financial investment and political leadership. These include

**Figure 3:**
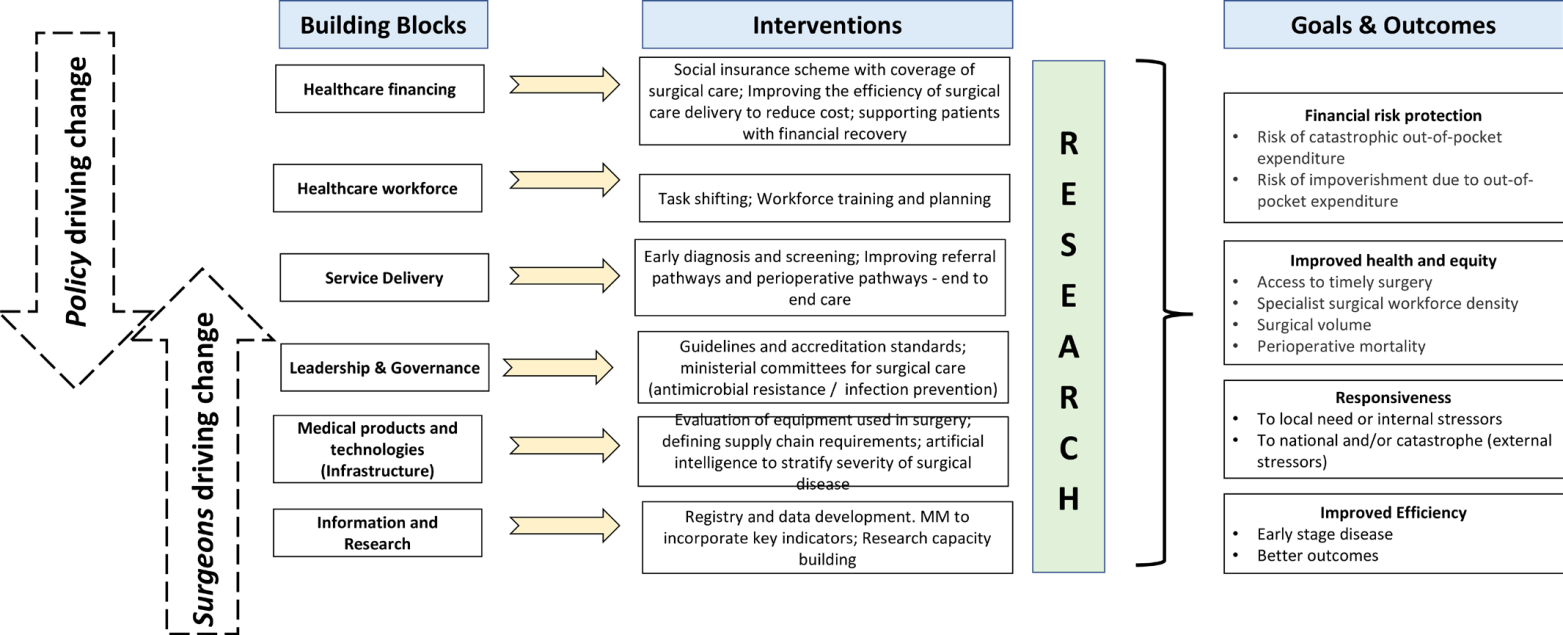
Health system building blocks, interventions and impact of perioperative pathways.

1. *A robust referral network system:* this is important to ensure that there is a timely referral from the community into first referral hospitals^38^ and also from the district hospitals into the tertiary centres for more complex care. Several nations have established a devolved system of healthcare governance in which authority, responsibility and financial resources are redistributed to different levels of government.^39^ This has the advantage of moving health services closer to the people, enabling referral systems and potentially lowering overall costs. It does however introduce challenges in implementing large infrastructure investment. Thus, strategic healthcare organisational strategies must be employed for high-complexity and low-acuity interventions to be referred to regional centres (regionalised), with common emergency and essential surgical interventions addressing low complexity and high acuity interventions available at district hospitals (decentralised) while employing a highly organised and streamlined referral system (figure 3).^26^ A streamlined referral system is required from the community to district hospital, as well as from district into regional centre. Strengthening these pathways of care can promote earlier access to treatment and better patient outcomes, thereby benefitting the entire health system.
2. *Improving surgical capacity and preparedness:* this requires training of specialist nurses, anaesthetists, as well as surgeons.^40 41^ District-level real-time granular data are still required for these strengthening efforts to assess relevance and impact. Global surgery and perioperative care networks are ready and able to support such assessments. For instance, the surgical preparedness index^42^ could be useful for gauging system readiness to provide effective and sustainable surgical care. This index, recently developed with 1632 hospitals in 119 countries, demonstrated variation in preparedness of hospital systems in external shocks such as the COVID-19 on elective surgical activity. Many lessons were learnt through this pandemic, which requires comprehensive planning and preparation for the future. An example would be identifying segregated care pathways or sites and workforce to continue or maintain elective surgical activity.^43^
3. *Upscaling quality and safety:* this is a particular concern if a rapid expansion of services is instigated. Poor-quality surgery can cause more harm than good. The G4 Alliance 11 Best Practice Recommendations for Quality Safe, Surgery, and Anaesthesia Care^25^ focus on building systems of safety that have been shown to be feasible and cost-effective in LMICs. A recent systematic review highlighted that implementation of evidence-based interventions in surgery and perioperative care within LMICs is limited.^22^ An example is the implementation of the WHO surgical safety checklist,^44 45^ where uptake is variable outside research settings. Therefore, there is a great need to scale this further to improve equity of care received by patients globally.

**Figure 4:**
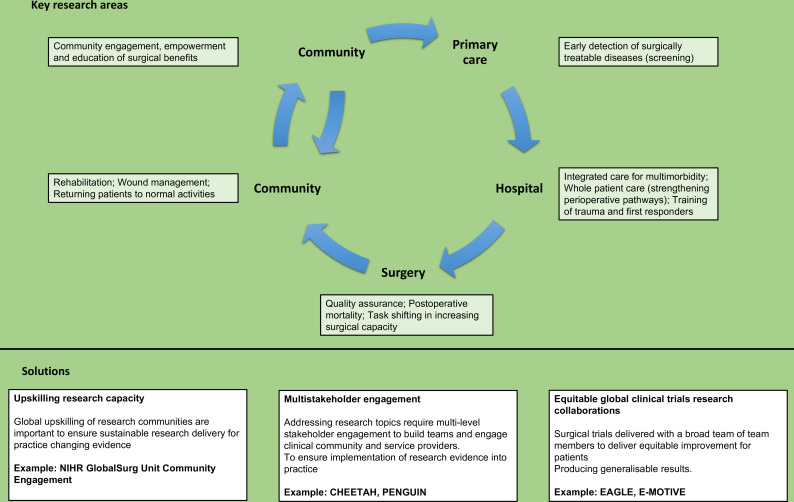
Key research areas and solutions for future research.

These challenges correspond to the WHO health systems strengthening building blocks seen through a global perioperative care lens (figure 4). Policy development in whole health systems needs to be informed by high-quality evidence which can now be collected across the surgical and perioperative care networks, assessing current surgical capacities, standardisation of care, effective governance and reported through prospective contemporaneous patient outcomes data.

#### Financing models

**Figure 5:**
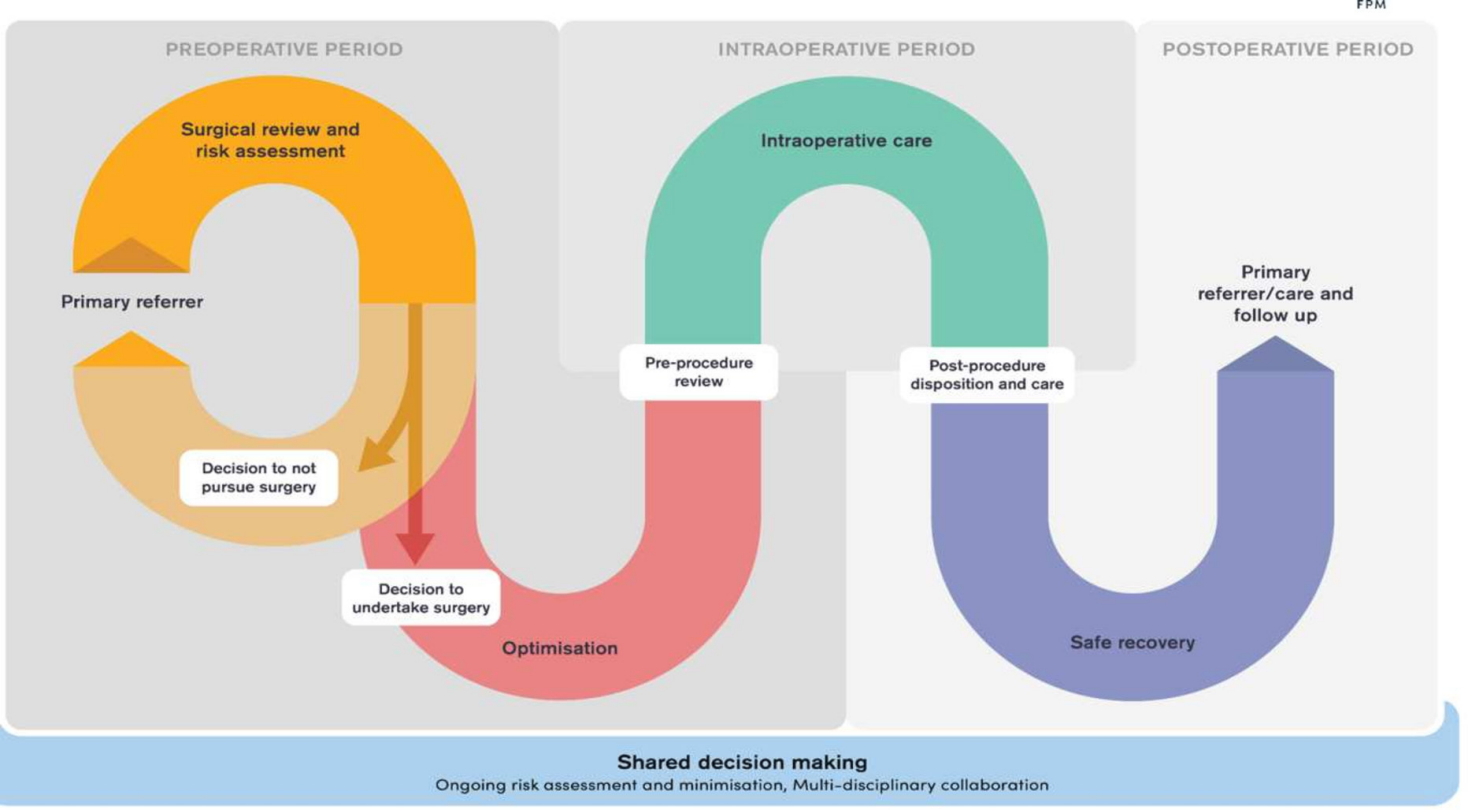
Model of perioperative care pathway.

Operationalisation of surgical and perioperative care pathways (figure 5) plans requires significant financial investment.^46^ This remains a major challenge. Current estimates for the cost of surgical system strengthening are staggering, ranging from US$69.7 million in Rwanda to US$16.8 billion in Nigeria.^47 48^ Financing these initiatives is complex and highly dependent on each country’s political commitment to health, income level, existing health financing system and relationships with external partners such as development banks, philanthropy and non-governmental organisations. Successful financing requires context-specific solutions at regional, national and local levels. These depend on the country’s ability to identify shared interests and strategically design sustainable models for whole health system strengthening.

Although a dedicated funding package that addresses the total costs of the National Surgical Plans (NSPs) may be a preferred option, the fiscal constraints and limited implementation capacity at the health ministries make this unlikely. Zambia’s experience illustrates how a piecemeal approach may be useful. For example, Zambia commenced surgical subspecialty training programmes immediately following the launch of their NSP, with graduates now entering the surgical workforce. Crucially, Zambia has established a national health insurance scheme^49^ for the formal sector which included a package of surgical care. This enables strengthening of the whole healthcare system with surgery as a component of this process. However, at this stage of development, the overall capacity to deliver surgical care remains constrained.^50 51^ A two-tiered system consisting of a non-revenue generating public system for the poorest and a revenue-generating system for those who can pay may offer the combination of equitable access to essential surgical care that is financially sustainable and potentially accelerate the service development. For instance, Rwanda has demonstrated the potential of such systems by using donor health funding strategically to enhance access.^48^

In 2001, Member States of the African Union committed to spending 15% of their national budget on health, known as the ‘Abuja Declaration’.^52^ However, meeting this target has been elusive but development of the national health systems to deliver equitable care can encompass surgery. For some countries, a private–public partnership model may serve to increase surgical service delivery while attracting private investments into the health sector. For example, a blended financing model^53^ combines public and donor investments with private capital to finance revenue-generating enterprises that can deliver affordable surgical care to neglected patients while generating sufficient revenues to be financially sustainable. This model can leverage concessional catalytic capital (grants) to unlock private capital, often several times the amount of grants.^54^

The discussion on financing must be contextualised within the historical and ongoing economic relationships that exist. It is crucial to acknowledge the extraction of resources from LMICs during colonisation and the ongoing unequal neocolonial economic relationships.^55^ The solution to global surgery should not rely solely on private capital, as this can be dangerous and misleading. Instead, fairer trade agreements and reparations should be considered. The health of citizens in the Global South should not be material for private investment and generating returns. A discourse focusing on private capital further advances the interests of Global North companies and industry at the expense of Global South citizens.^55^

The substantial societal cost of inadequate surgical care needs emphasis, especially in emergency care systems. One study showed the cost of not providing treatment for appendicitis alone is costing some countries more than 1% of GDP.^56^ Providing the clinical capacity to deliver care for emergency surgical diseases will substantially strengthen the wider health system by improving emergency response services. Considering surgery as an integral part of the health system can enable further investment and wider improvements to healthcare.^46^ By 2030, we hope to see LMICs funding their own NSOAPs and demonstrating improved access. An example of an LMIC funding greater access itself, even if it is the recipient of donor health funding, is Rwanda, which has strategically used donor funds to improve surgical access. This demonstrates the potential for LMICs to take ownership of their health financing and improve access sustainably.

### Emerging solutions

We are halfway in time towards the 2030 SDGs. It is appropriate to now consider how to achieve these targets. One critical action, initiated within the recent WHA resolution and outlined in this and the accompanying papers, is to position surgery within the compass of health system strengthening. This emphasises the key role of surgery in the management of multimorbidity and NCDs and demonstrates how surgery adds resilience to the health system, defining it as a central part of sustainable health systems. This realignment critically allows expenditure on surgical services not to be seen in isolation, but as an investment in the health systems as a whole.

Current service shortfalls must be addressed through strengthening care pathways around district hospitals, not just tertiary centres. Rapidly expanding these district hospital services should be a priority. This clearly requires much more than simply training more surgeons, a target that is even being missed even in some high-income countries. Development of allied health specialties will be required to deliver accelerated expansion of services, encompassing the whole care pathway. Training programmes, aligned with local district hospital needs, for surgeons and allied health disciplines need to be developed and propagated within coordinated programmes of south-to-south learning. Successes in one region should be explored across a wider geography.

A comprehensive systems approach should address end-to-end management from surveillance, prevention, prehospital care, surgical care, to rehabilitation. The six WHO health system building blocks can be used to help achieve this comprehensive systems approach (figure 4). However, this will require strong leadership from both surgical teams and policy-makers. The existing research evidence to support investment in perioperative care is limited and requires urgent attention^57^ (figure 5). However, evidence for investment in NCDs (such as cancer) is growing and will require development of peri/operative care services within their compass. High-quality contemporaneous research evidence identifying improved patient outcomes will support and drive this policy change.

There is a risk associated with rapid expansion of surgical services, namely an associated reduction in quality and a rise in adverse patient outcomes. Once again, it will be imperative that patient-level assessment of outcomes is collected to ensure we maintain and improve standards of care. This research activity can also promote south-to-south learning and engage the wider surgical community in this essential service-strengthening programme. High-quality research can be driven by the surgical and perioperative care community and investigating the whole pathway of end-to-end care will be essential to develop and maintain these standards. Real-time patient-level data, collected at the district level, ideally (and efficiently) provided through global snap-shot audits, can be used to assess uptake of innovation and impact on patient outcomes. The surgical community across the world has shown that it can be engaged in driving these critical healthcare developments.

## Data Availability

There are no data in this work.
